# *N*-Cyanomethylnorboldine: A New Aporphine Isolated from *Alseodaphne perakensis (Lauraceae)*

**DOI:** 10.3390/molecules16043402

**Published:** 2011-04-20

**Authors:** Mohd Azlan Nafiah, Mat Ropi Mukhtar, Hanita Omar, Kartini Ahmad, Hiroshi Morita, Marc Litaudon, Khalijah Awang, A. Hamid A. Hadi

**Affiliations:** 1 Chemistry Department, Faculty of Science and Mathematic, University of Pendidikan Sultan Idris, Tg. Malim, Perak, Malaysia; E-Mails: azlan@upsi.edu.my (M.A.N.); kartini@fsmt.upsi.edu.my (K.A.); 2 Centre for Natural Products and Drug Discovery, Block D, Department of Chemistry, Faculty of Science, University of Malaya, 50603 Kuala Lumpur, Malaysia; E-Mails: matropi@um.edu.my (M.R.M.); khalijah@um.edu.my (K.A.); hanita74@um.edu.my (H.O.); 3 Faculty of Pharmaceutical Sciences, Hoshi University, Ebara 2-4-41 Shinagawa, Tokyo 142-8501, Japan; E-Mail: moritah@hoshi.ac.jp (H.M.); 4 Centre de Recherche de Gif, Institut de Chimie des Substances Naturelles, CNRS, 1, Avenue de la Terrasse, 91198 Gif-sur-Yvette Cedex, France; E-Mail: marc.litaudon@icsn.cnrs-gif.fr (M.L.)

**Keywords:** *Alseodaphne perakensis*, *Lauraceae*, aporphine alkaloid

## Abstract

A phytochemical study of the bark of *Alseodaphne perakensis* has yielded three aporphine alkaloids: the new compound *N*-cyanomethylnorboldine (**1**), and the two known alkaloids *N*-methyllaurotetanine (**2**) and norboldine (**3**)**.** The isolation was achieved by chromatographic techniques and the structural elucidation was performed via spectral methods, notably 1D- and 2D-NMR, UV, IR, and HRFABMS. The vasorelaxation activity of compound **1 **has been studied.

## 1. Introduction

**Figure 1:**
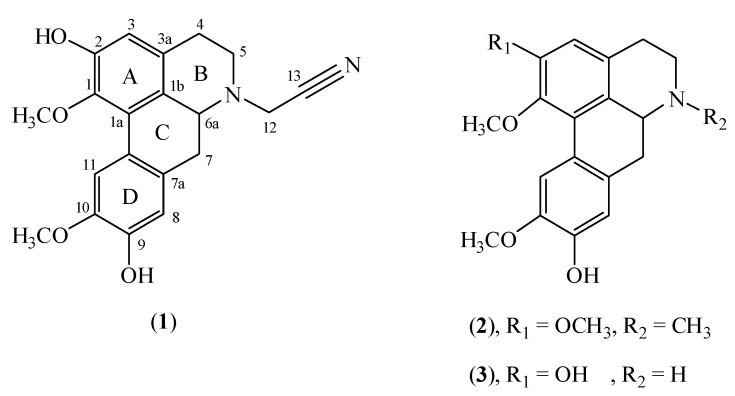
Alkaloids **1**-**3** isolated from *Alseodaphne perakensis*.

*Alseodaphne perakensis* (Gamble) Kosterm from the family *Lauraceae* is a small tree of about 6 meters in height and with yellow-green flowers, distributed mainly in Peninsular Malaysia. *Alseodaphne* species have been reported to contain bisbenzylisoquinolines [1] and oxobisbenzyl-isoquinolines [2]. In this paper, we report the isolation and characterization of a new aporphine alkaloid, *N*-cyanomethylnorboldine (**1**), together with the known aporphines *N*-methyllaurotetanine (**2**) and norboldine (**3**) (Figure 1) from the bark of *Alseodaphne perakensis* collected at Sg. Merantor, Gua Musang, Kelantan, Malaysia. 

## 2. Results and Discussion

**Figure 2:**
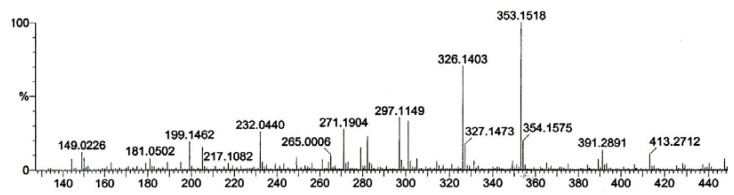
HRFABMS Spectrum of alkaloid **1**.

Compound (**1**) was obtained as a yellowish amorphous solid with [α]_D_^26^ -16.5^o^ (*c* 0.5, MeOH). The HRFABMS (Figure 2) exhibited the [M+H]^+^ peak at *m/z* 353.1518 (calc. 353.1501) thus suggesting a possible molecular formula C_20_H_20_N_2_O_4_. Furthermore, the IR spectrum showed the presence of O-H and -CN stretching at 3,348 and 2,250 cm^-1^, respectively.

**Figure 3:**
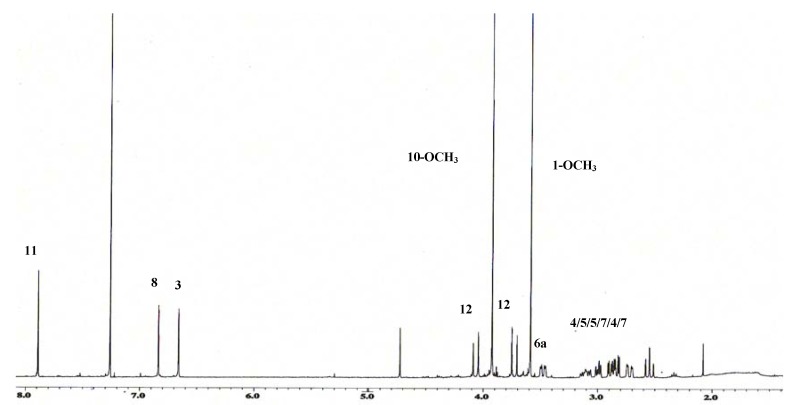
^1^H-NMR spectrum of alkaloid **1**.

The ^1^H-NMR spectrum (Figure 3) exhibited the signals of three aromatic protons at δ 6.66, 6.83 and 7.89 which correspond to H-3, H-8 and H-11, thus indicating the compound was an aporphine alkaloid [3]. The NMR spectrum further revealed two methoxyl singlets at δ 3.58 and 3.92, which correspond to C-1 and C-10, respectively. A doublet of doublets appeared at δ 3.48 (*J* = 13.7 and 4.1 Hz) which was attributable to H-6a. A doublet signal appearing at δ 3.73 (*J* = 17.8 Hz) was coupled with the doublet resonances at δ 4.06 (*J* = 17.8 Hz) which were part of the geminal proton system at C-12. In addition, three doublets of doublets were observed at δ 2.72 (*J* = 13.2 and 3.2 Hz), δ 2.89 (*J* = 12.3 and 3.6 Hz) and δ 2.83 (*J* = 13.7 and 4.1 Hz) which were attributable to H_β_-4, H_α_-5 and H_β_-7, respectively. Moreover another proton at H_α_-7 resonated at δ 2.54 as a doublet of triplets (*J* = 13.7 and 0.9 Hz).

The NOE differential spectrum was used to confirm the position of the methoxyl groups on the A and D rings. Detailed analysis on the NOE experiment showed that irradiation of H-11 (δ 7.89) led to a 8.74% and 2.07% enhancement of the methoxyl groups at C-10 (δ 3.92) and C-1 (δ 3.58), respectively, while irradiation of H_β_-12 (δ 4.06) showed an enhancement of doublet signal at δ 3.73 (12.81%). This confirmed that C-12 was substituted by a cyano group.

**Table 1:** ^1^H-NMR (400 MHz) and ^13^C-NMR (100 MHz) spectral data of compound **1 **in CDCl_3_ (*δ *in ppm, *J *in Hz).

Position	δ^ 1^H ( *J*, Hz)	δ^ 13^C	HMBC (^2^ *J*, ^3^*J*)	COSY
1		142.1		
1-OCH_3_	3.58 ( *s*)	60.4		
1a		126.1		
1b		125.7		
2		148.3		
3	6.66 ( *s*)	113.2	1,1b,2,4	
3a		129.3		
4	3.11 ( *m*)	28.8	3a,5	H_5_
	2.72 ( *dd*,16.4,3.2)			
5	2.89 ( *dd*,12.3,3.6)	50.4	3a,6a	H_4_
	3.00 ( *dd*,11.4,5.9)			
6a	3.48 ( *dd*,13.7,4.5)	58.0		H_7_
7	2.54 ( *ddd*,13.7,0.9)	33.7	1b,6a,7a,8	H_6a_
	2.83 ( *dd*,13.7,4.1)			
7a		128.9		
8	6.83 ( *s*)	114.4	7,10,11a	
9		145.2		
10		145.8		
10-OCH_3_	3.92 ( *s*)	56.2		
11	7.89 ( *s*)	110.1	7a,9,1b	
11a		123.4		
12	3.73 ( *d*,17.8)	43.3	5,6a,13	
	4.06 ( *d*,17.8)			
13		114.3		

**Figure 4:**
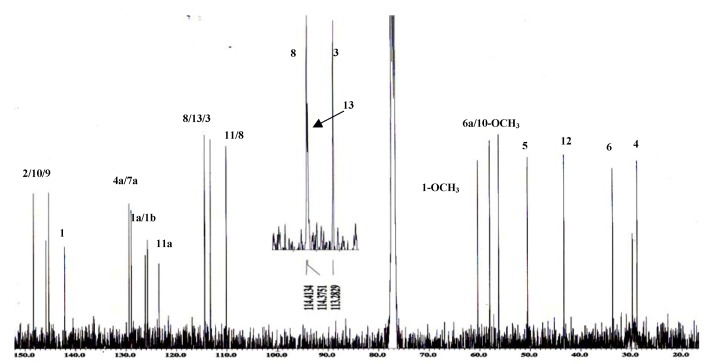
^13^C-NMR spectrum of alkaloid **1**.

The ^13^C-NMR spectrum (Figure 4) showed all twenty carbons in the molecule and showed two signals at δ 56.2 and 60.4 belonging to 10-OCH_3_ and 1-OCH_3_, respectively. In addition, a signal at δ 58.0 was attributable to C-6a. Four methylene carbons were observed at δ 28.8 (C-4), 33.7 (C-α), 43.3 (C-12) and 50.5 (C-5). A signal for a carbon attached to a nitrogen atom was observed at δ 114.3.

**Figure 5:**
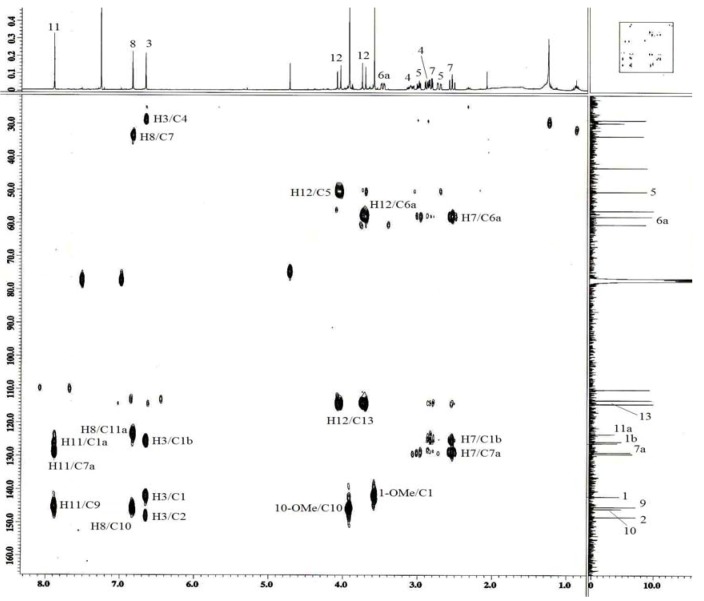
HMBC-NMR spectrum of alkaloid **1**.

**Figure 6:**
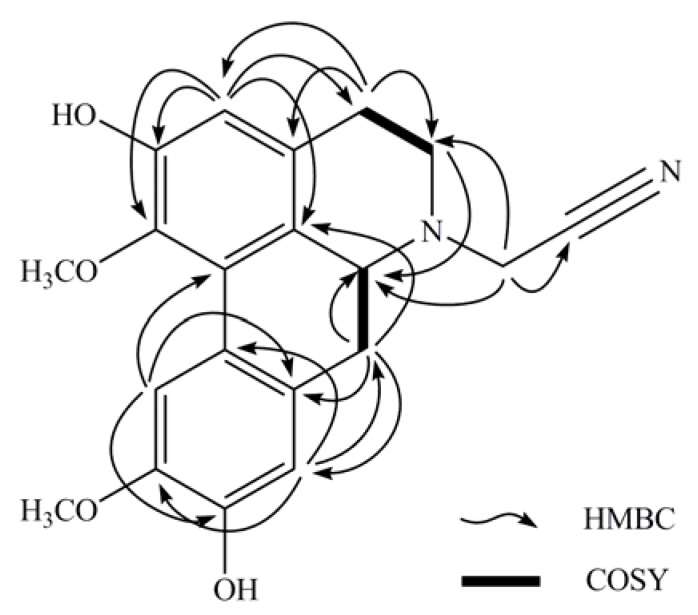
Selected 2D NMR correlations of *N*-cyanomethylnorboldine (**1**).

The HMBC experiment (Figure 5 and Figure 6) further confirmed the structure of alkaloid **1**. In the spectrum, cross-peaks were found for H-3 (δ 6.66) with C-1 (δ 142.1), C-2 (δ 148.3), and C-1b (δ 125.7), H_α_-12 (δ 3.73) with C-5 (δ 50.4) and H_β_-12 (δ 4.06) with C-6a (δ 58.0). 

**Figure 7:**
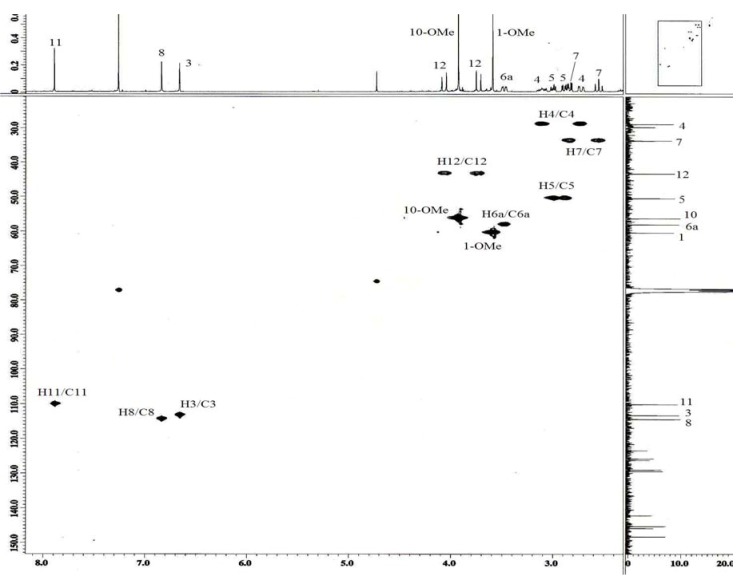
HSQC-NMR spectrum of alkaloid **1**.

The above mentioned data and that from the HSQC spectrum (Figure 7) confirmed that the two methoxyl groups were positioned at C-1 and C-10.

The new compound **1** (at 10^-4^ M) showed no effective vasorelaxant effects on rat aorta strip induced contractions using a reported procedure [4], although some alkaloids have been reported to show such vasodilation activity [1,2].

The identification of the *N*-methyllaurotetanine (**2**) [5,6,7] and norboldine (**3**) [8,9,10,11], and determination of stereochemistry was accomplished by comparisons of UV, IR, MS, ^1^H-NMR, and ^13^C-NMR with data reported previously for these compounds.

## 3. Experimental

### 3.1. General

Optical rotations were determined on a Jasco (Japan) P1010 instrument equipped with a tungsten lamp. Mass spectra were obtained on a Jeol JMS 700 TZ spectrometer using *meta*-nitrobenzylalcohol (NBA) or glycerol as the matrix for FAB analysis. The NMR (^1^H-, ^13^C- and 2D-) spectra were recorded in deuterated chloroform on a JEOL 400 MHz instrument. Chemical shifts were reported in ppm on δ scale and the coupling constants were given in Hz. Glass and aluminium supported silica gel 60 F_254_ plates were used for Thin Layer Chromatography (TLC). TLC spots were visualized under ultraviolet light (254 and 365 nm) and by spraying with Dragendorff’s reagent. Silica gel 60, 70-230 mesh ASTM (Merck 7734) and silica gel 60, 230-400 Mesh ASTM (Merck 9385) were used for column and flash chromatography, respectively.

### 3.2. Extraction and Isolation of the Alkaloids

A voucher specimen (KL 5135) has been deposited at the Herbarium of the Chemistry Department, Faculty of Science, University Malaya, Kuala Lumpur, Malaysia. The dried, ground bark (3.0 kg) was first defatted with hexane (6.0 L) at room temperature for 60 hours. The residual plant material was dried and left for 2 hours after moistening with 25% NH_4_OH. It was then re-extracted with CH_2_Cl_2_ (11.0 L) in a Soxhlet extractor for 17 hours. After filtration, the supernatant obtained was concentrated to 500 mL, followed by acidic extraction with 5% HCl until a negative Mayer’s test was obtained. The aqueous solution obtained was made alkaline (pH 11) with NH_4_OH and re-extracted with CH_2_Cl_2_. This was followed by washing with distilled H_2_O, drying over anhydrous sodium sulphate, and evaporation to give the alkaloid fraction (10 g). The crude alkaloid (5.0 g) was subjected to column chromatography over silica gel using CH_2_Cl_2_ gradually enriched with methanol as eluent. The isolated fractions were subjected to column chromatography on silica gel with CH_2_Cl_2_-MeOH (90:10) solvent system followed by preparative TLC with CH_2_Cl_2_-MeOH (95:5) solvent system to give *N*-cyanomethyl-norboldine) (**1**, 8 mg), *N*-methyllaurotetanine (**2**, 11 mg) and norboldine (**3**, 15 mg). 

*N-cyanomethylnorboldine* (**1**): Isolated as a yellowish amorphous solid; HRFABMS [M+H]^+^* m/z* 353.1518 (calc. 353.1501); UV (MeOH) λ _max_: 310; IR (CHCl_3_) cm^-1^: 3392 (OH) and 2250 (CN); ^1^H-NMR and ^13^C-NMR (CDCl_3_) ppm: see Table 1.

### 3.3. Vasorelaxant Activity

The vasorelaxant effects bioassay was carried out using induced rat aorta ring contractions. The thoracic aortas were removed from 10 weeks old Wistar rats sacrificed by bleeding from the carotid arteries under anesthetization. A section of the thoracic aorta between the aortic arch and the diaphragm was removed and placed in oxygenated, modified Krebs-Henseleit solution. The aorta was cleaned of loosely adhering fat and connective tissue and cut into ring preparations 3 mm in length. The tissue was placed in a well-oxygenated (95% O_2_, 5% CO_2_) bath of 10 mL KHS solution at 37 °C with one end connected to a tissue holder and the other to a force-displacement transducer (Nihon Kohden, TB-611T). The tissue was equilibrated for 60 min under a resting tension of 1.0 g. During this time the KHS in the tissue bath was replaced every 20 min. After equilibration, each aortic ring was contracted by treatment with 3 × 10^-7 ^M norepinephrine (NE). The presence of functional endothelial cells was confirmed by demonstrating relaxation to 10^-5^ M acetylcholine (Ach), and aortic ring in which 80% relaxation occurred, was regard as tissues with endothelium. The endothelial cells were removed by gentle rubbing and were confirmed by loss of Ach-induced relaxation. When the NE-induced contraction reached plateau, each sample was added to the bath.

## 4. Conclusions

To our knowledge, this is the first report on the occurrence of *N*-cynomethylnorboldine (**1**) in the *Alseodaphne* species. This new aporphine has no vasorelaxant activity on induced contractions of rat aorta rings.
